# (In)effective realism?

**DOI:** 10.1007/s13194-021-00441-x

**Published:** 2022-04-25

**Authors:** Juha Saatsi

**Affiliations:** School of Philosophy, Religion and History of Science, University of Leeds, Leeds, England

**Keywords:** Scientific realism, Effective ontology, Matthias Egg, Quantum mechanics

## Abstract

Matthias Egg (2021) argues that scientific realism can be reconciled with quantum mechanics and its foundational underdetermination by focusing realist commitments on ‘effective’ ontology. I argue in general terms that Egg’s effective realism is ontologically overly promiscuous. I illustrate the issue in relation to both Newtonian mechanics and quantum mechanics.

## Introduction

Scientific realism is notoriously problematic in connection with quantum mechanics. A key problem is underdetermination: there are many competing formulations of quantum mechanics at the foundational level, so it is unclear what we should be realists about. Matthias Egg (2021) argues that scientific realism can be reconciled with quantum mechanics by articulating realist commitments in terms of “effective” ontology.^1^ Egg argues that a realist interpretation without undue metaphysical speculation can be given of “Textbook Quantum Mechanics” (TQM), that is, quantum mechanics “as it is formulated in standard textbooks.” Egg argues that despite its “somewhat messy and recipe-like form” TQM can be construed as an “effective theory” the ontology of which is given “in *functional* terms [and not by] reference to the *nature* of their theoretical posits.” (p. 7, original emphasis)

I will argue in general terms that Egg’s conception of “effective” (or “functional”) ontology is ontologically overly promiscuous. More specifically, I will argue that it is in tension with how physics deals with theoretical inconsistencies by adjudicating

ontological commitments in a more fine-grained manner. To the extent Egg’s novel conception of realist ontology is thus problematic, it does not salvage realism about quantum mechanics either.

The idea that realist commitments should focus on “effective” ontology has become increasingly popular. Different versions of the broad idea can be found in, e.g., Ladyman and Ross (2007), Wallace (2012), Williams (2019), Fraser (2018), Fraser (2020), and Cordero (2020). Here I will focus specifically on Egg’s articulation of this idea in the broader context of scientific realism. I will begin by reviewing Egg’s argument (Section 2), before arguing that it problematically reifies theoretical inconsistencies (Section 3). I will illustrate the issue at stake with reference to Newtonian forces, indicating how effective ontology clashes with the scientific aim of working out realist commitments in a consistent manner in the light of the big picture (Section 4). Finally, I will examine the issue in connection with TQM (Section 5).

## Egg on underdetermination and effective ontology

Egg defends a “non-fundamentalist approach to ontology” in response to the problem that there is “a considerable number of ontological options compatible with the empirical basis of QM” (p. 3). How could a scientific realist justify a choice between the alternative ‘interpretations’ quantum mechanics — Everettian QM, spontaneous collapse theories, Bohmian mechanics, etc. — without indulging in metaphysical speculation that transcends the evidential basis that realism epistemologically relies on? This challenge is an instance of the problem of underdetermination of theory by evidence, a well-known problem to scientific realism (Acuña, 2021; French and Saatsi, 2020). Egg nicely presents the challenge as involving two horns:

[E]ither opt for one particular version of QM, but then you face the above-described problem of underdetermination; or limit your ontological commitment to some core content of QM unaffected by the underdetermination between its different versions, but this seems to lead you back to the somewhat anti-ontological “Copenhagen” way of thinking about QM. (p. 3)

So, on the one hand, one can articulate the ontology of QM on the basis of this or that formulation/interpretation of QM (as philosophers of physics do), but then one cannot justify a scientific realist attitude towards it due to the problem of underdetermination. On the other hand, one can avoid the underdetermination by limiting one’s realist commitments to what is shared by all the alternative formulations, but then it is unclear “whether the resulting commitments are substantive enough to constitute an interesting quantum ontology” (p. 5)

Egg argues that a substantive enough quantum ontology, one that is not threatened by underdetermination, can actually be found in TQM. The key is to acknowledge that the latter is an *“effective theory”*. The distinction between between effective and fundamental theories underwrites Egg’s demarcation between effective and fundamental ontology. As to the former distinction, “what marks a theory as effective is that it successfully deals with all the phenomena within a natural domain, where ‘natural’ is usually spelled out in terms of a physical parameter (energy, for example) which needs to be in a certain range for the theory to be applicable.” (Egg, 2021, p. 8) For example, Newtonian mechanics is an effective theory since it is successfully applicable in the non-quantum domain of low relative speeds and weak gravitational fields, and non-relativistic quantum mechanics is an effective theory as a low-energy limit of quantum field theory.

Effective ontology is just the ontology of effective theories. According to Egg, an effective theory’s ontology is whatever theoretical posits “actually perform the explanatory and predictive work” in such a theory (p. 22):

But what is the content of such an ontology? In the absence of a proper solution to the measurement problem, does TQM provide us with anything over and above a mathematical formalism and some rules on how to extract empirical predictions from it? To see how it does, one needs to realize that questions about the ontology of effective theories must be answered in *functional* terms. They cannot be answered by any reference to the nature of their theoretical posits, insofar as that would require knowledge about how these posits emerge from a fundamental theory, which is just what the effective theory does not provide. Instead, the posits of an effective theory are characterized by what they *do* (effectively), rather than by what they *are* (fundamentally). (p. 7, original emphasis)

This encapsulates Egg’s account of effective ontology. He elaborates on these general remarks by giving some specific examples, advocating for instance “realism about spin states (and their components) insofar as they are assigned to quantum systems by TQM.” (p. 12) (I will examine the case of spin in Section 5.)

So much for Egg’s conception of effective ontology. The main problem I see with it is that it turns out to be overly promiscuous for scientific realism. Before I argue for this, I want to make it clear that I am *not* suggesting that scientific realists should be (only) committed to fundamental ontology. Indeed, while the distinction between fundamental and non-fundamental ontology is important in e.g. contemporary metaphysics and philosophy of physics, it arguably has not been central to scientific realism. Looking at the scientific realist literature, one can see that most realists commit to the reality of molecules, atoms, bacteria and viruses, and other things that are obviously not fundamental (whatever that means exactly). Furthermore, arguably a realist attitude towards such non-fundamental posits should not require realism about their more fundamental ‘constituents’. After all, it should be possible to find out about reality of molecules, for example, before finding out about electrons, say. Indeed, it seems that a realist epistemology of science couldn’t get off the ground at all if we did not allow ontological commitment to such non-fundamental features of the world, for we have no well-confirmed scientific theories about the *truly* fundamental. So, from the point of view of the scientific realism debate a realist commitment to non-fundamental ontology thus seems incontrovertible, and scientific realists who equate ‘real’ with ‘fundamental’ are rare outside philosophy of physics.^2^

Realists at large thus agree with Egg’s positive attitude towards non-fundamental ontology. This does not mean that realists should agree with Egg’s realist commitment to effective ontology, however. The latter saddles the realist with principles of ontological commitment — a novel meta-ontology — according to which scientific realists should indiscriminately commit to whatever theoretical posits are ‘doing the work’ in our best scientific theories (in the sense indicated above). This meta-ontology can be compared with other ontological frameworks that scientific realists have adopted. For example, a prominent meta-ontological stance amongst realists involves regarding theoretical posits’ *explanatory* contributions as indicating ontologically commitment. Such explanatory contributions necessarily involve ‘doing’ (as opposed to just ‘being’) in the sense of causing or being otherwise *explanatorily responsible* for empirical phenomena. But, notably, realist analyses of scientific explanations’ ontological commitments has nothing to do with the distinction between fundamental and non-fundamental, since good (accepted, well justified, etc.) scientific explanations can be furnished by fundamental and effective theories alike. Moreover, arguably the ontological commitments induced by explanatory indispensability are best analysed on a *case-specific basis* that does not lend itself to an overarching meta-ontological thesis (Saatsi, 2018). Sometimes indispensable explanatory posits are ontologically committing, sometimes not, and a lot depends on the details: what kind of explanation is at stake, how it explains, and how the explanation in questions hangs together with the rest of science. For example, despite the various explanatorily effective roles played by idealisations, mathematics, and non-mathematical abstract objects (cf. Psillos, 2010), it has been argued that such indispensability does not imply a realist commitment, all things considered (see, e.g., Saatsi, 2016a,??).

In sum, in response to the underdetermination challenge with respect to quantum mechanics Egg is proposing that scientific realists should think of ontological commitment in a particular way. We should evaluate this novel meta-ontology in the context of scientific realism in general terms before examining it in connection with TQM. To this end I will next argue that due to inconsistencies in the ensuing ontological commitments realists are better off not committing to the theoretical posits of all effective theories in such an undiscriminating manner.

## Tensions arising

According to Egg “the very idea of non-fundamental ontology requires some defense.” While this is certainly right as far as certain metaphysical debates are concerned (e.g., involving ontological nihilism), I have already indicated that it is not clear how this connects to scientific realism literature. As noted, realists at large have not viewed non-fundamental ontology of science requiring any kind of *general* defence. Realists have happily defended realism about atoms, molecules, viruses, and such without any qualms about their non-fundamentality. Scientists provide good evidence for them, they explain by appealing to them, and they don’t regard them in tension with the more fundamental areas of science. It would be extremely un-naturalistic for realists to deny the reality of, say, viruses, due to them not being fundamental.

Non-fundamental ontology thus seems an uncontested and unproblematic part of the everyday and scientific image as typically presented within the realism debate. (Only analytic metaphysicians worry about the reality of apples or viruses as non-fundamental.) But accepting non-fundamental ontology in this spirit is far from adopting Egg’s effective ontology. The latter, as we have seen, is characterised in abstract terms by reference to ‘functions’ and ‘doings’. The problem with effective ontology is that it ends up containing more than realists should want to bargain for. Construing the indispensable theoretical posits of all effective theories realistically commits one equally to, e.g., light rays (and also rays in geophysics, etc.) given the effectiveness of geometrical optics, and Newtonian gravitational force (and Coriolis force and other ‘pseudo-forces’), given the effectiveness of Newtonian physics. Such theoretical notions are indispensable to a good effective theory, but indiscriminating commitment to their reality leads to serious theoretical tensions arising from inter-relations between more and less fundamental theories, and also from within a given scientific theory, as we will see.

Such tensions animate wide-ranging metaphysical debates on reduction and emergence. Here are two examples of widely held views that speak against effective ontology:

(1) Geometrical ‘ray’ optics is inconsistent with the wave theory of light: it is prima facie incoherent to maintain that light *is* a ray, and that it also *is* a wave. There are, of course, aspects of light waves, as described by wave optics, that can be associated with a theoretical description of light in terms of rays. This inter-theoretic relationship is much studied and well understood (see, e.g., Kline & Ray, 1965). From the perspective of wave optics light rays are not real, but geometrical optics is nevertheless “approximately true”: the predictive and explanatory effectiveness of geometrical optics can be precisely accounted for in wave-theoretic terms. This resolves the tension.

(2) Newtonian gravity is inconsistent with General Theory of Relativity: it is prima facie incoherent to maintain that gravity is a Newtonian force, and that it is also a manifestation of spacetime’s curvature and geodesic (force-free) motion therein. There are, of course, aspects of general relativistic dynamics that can be associated with the Newtonian description of gravity. This underwrites an inter-theoretic relationship between the two theories that is much studied and overall well understood (see, e.g. Malament, 2012). From the relativistic perspective Newtonian gravitational force is not real, but Newton’s theory can nevertheless be regarded as “approximately true”: its predictive and explanatory effectiveness can be precisely accounted for in general relativistic terms. This resolves the tension.

Both of these views can be contested, of course. Some have argued, for example, that some aspects of optical phenomena require a more serious commitment towards a ray-like description of light than can be underwritten by a purely wave-theoretic perspective (e.g., Batterman, 2001, see Pincock, 2011 and Belot, 2005 for critical discussion). Egg likewise argues that Newtonian forces should be part of the realist’s effective ontology. I will discuss Egg’s argument below, but here’s the general point. Reflecting on these kinds of inter-theoretic relations (and the associated debates regarding reduction and emergence) indicates how a blunt appeal to effective ontology results in theoretical tensions that *both philosophers and scientists* have been keen to relieve, often by carefully articulating or explaining away tension-inducing ontological commitments in order to achieve overall theoretical coherency. Where no such tension exists, no articulation or elimination is called for. I take it that no tension exists between fundamental physics and our scientific commitment to the COVID-19 virus, its mechanisms of replication and propagation, for example. Realism about the non-fundamental in and of itself has nothing to do with whether or not we deem science as having discovered that there are no light rays (despite the ‘effective ontology’ of geometrical optics); no Newtonian gravitational force (despite the ‘effective ontology’ of Newtonian theory); etc. Viruses can still be real — as real as apples. Instead of arguing for effective realism (or some other general meta-ontological view) as a part of scientific realism, our efforts are better spent on contributing to the scientific image by recognising and relieving such tensions where they exist.

Incoherency-inducing theoretical notions can be altogether eliminated from ontology, or their ontological status can be relegated, by accounting for their theoretical usefulness in effort to achieve an overall consistency in the scientific image. Sometimes this works from the perspective of a more fundamental theory, and sometimes from within a single theoretical framework. Arguably ontological elimination has been achieved for the two examples above. Importantly, such judgements do not require a general meta-ontological framework, since the theoretical details themselves can suffice to support the conclusion that some apparent ontological commitments are only that: apparent.

Effective ontology needs further defending wherever such theoretical tensions are manifested, not as an alternative to fundamental ontology, but to a more naturalistic ontological attitude that appreciates scientists’ achievements in making the overall scientific image more coherent.^3^ The meta-ontology of effective realism is problematic due to being in tension with scientists’ own reasoning about ‘what is real’, which is arguably also capable of accounting for the usefulness of theoretical posits that are not taken ontologically seriously. I will next elaborate on this by noting some subtleties regarding the ‘reality’ of forces.

## Forces or ‘fiction’?

Egg recognises the worry that “attributing substantive ontological content to effective theories […] might lead to an unacceptable proliferation of ontological commitments.” (p. 8) But he skirts around the heart of the issue, which I will now illustrate in detail.

After introducing effective realism Egg addresses the objection that “effective realism entails ontological commitment to gravitational forces, but general relativity tells us that gravity is not a force, hence effective realism is problematic.” (*ibid.*) As to what the problem is, Egg opines: “presumably [the] reason to resist realism about gravitational forces is that they are absent from our most fundamental theory of gravity (i.e., general relativity).” (p. 9) In response he notes that it is not only gravitational force that is absent from ‘fundamental’ physics’: the latter does not feature (e.g.) viruses either, “whose reality is well established, although they do not appear in fundamental physics.” (p. 10) In other words, the allegation is that realism about non-fundamental theoretical posits stands or falls with realism about gravitational forces: it’s effective ontology or bust for the realist.

This line of thought throws the baby of non-fundamental ontology out with the bathwater of effective ontology. The key distinction between viruses (and atoms, molecules, and apples…), on the one hand, and Newtonian gravitational forces, on the other, is that only the latter are flatly contradictory with the more fundamental theory in question. The issue is not that according to general theory of relativity (GTR) Newtonian gravitational theory is always strictly speaking false, only arising as an approximation at the limit of weak gravitational fields and velocities slow relative to the speed of light. Rather, the issue is that the Newtonian *concept* of gravitational force is inconsistent with GTR. The Newtonian concept is one that causes acceleration in an inertial frame of reference. In GTR, however, gravity comes out as conceptually equivalent to ‘fictitious’ inertial forces in Newtonian mechanics that only appear as a function of the system being described in an accelerating, non-inertial frame of reference. In GTR gravity can be always made to disappear in a local inertial frame (and there are no global inertial frames).

The point is simply that you can’t have it conceptually both ways: the features of the world that are required for the applicability of the Newtonian concept never occur according to GTR. This is analogous to how the absolute simultaneity of Newtonian mechanics is unreal according to special relativity. The practical applicability of absolute simultaneity — the sense in which it can be approximately true to say that two events are simultaneous for all of us — is explained by the relevant inter-theoretic relationship, but absolute simultaneity does not become ‘real’, or part of physics’ ontology. Physicists (at least if they are being careful and consistent) do not say that absolute simultaneity is a real feature of the world for objects moving ‘slowly’ relative to the speed of light. Nor do they say that Newtonian gravitational force is real in such-and-such settings, or at such-and-such a scale.

Egg says he “struggle[s] to understand how one can claim that general relativity somehow excludes the reality of gravitational forces without admitting that the reality of viruses is likewise ‘excluded’ by the standard model of particle physics.” (p. 10) I have already explained how the choice between mad-dog eliminativism and promiscuous effective realism is a false dilemma, but the notion of ‘gravitational force’ calls for further elaboration.

Denying the reality of *Newtonian* gravitational forces does not mean that there is no gravitational force in GTR. Rather, it means that the concept of gravitational force in GTR is inconsistent with the *Newtonian* gravitational force. GTR must accommodate, of course, e.g., the phenomenon of a material body experiencing ‘weight’ against the floor of a rocket ship that is (1) accelerating, or (2) suspended (as opposed to free-falling) in an appropriate gravitational field. GTR can do that by identifying how the relevant forces depend on the appropriate coordinate system and the total mass-energy of the body (see, e.g., Ridgely, 2010). From covariant divergence of the stress-energy-momentum tensor one can derive an expression for the ‘general force’ experienced by an observer in general coordinates. And from this expression one can derive forces in specific coordinate systems, such as a local co-moving coordinates of a uniformly accelerating observer, or the Schwarzschild coordinates in the case of a stationary observer in a spherically symmetric spacetime. These then yield equations that can be formally identified with Newton’s second law and Newton’s law of gravitation, at the appropriate limits of weak acceleration and weakly gravitating source, respectively (*ibid.*). The ‘general force’ equation similarly entails the forces experienced by observers due to rotating coordinates, corresponding to centrifugal and Coriolis forces. Critically, these derivations manifestly show how in *all* of these cases the force in question is on an equal ontological footing as an *inertial* one, to be contrasted with Newtonian gravitational force (*ibid.*). In the big picture, therefore, the force that Newton’s gravitational theory so effectively captures cannot be conceptually equated with the Newtonian gravitational force simply on pain of contradiction: gravitational force cannot be inertial and non-inertial at the same time.^4^

Could an effective realist maintain that both inertial and non-inertial gravitational force are real, one just being more fundamental than the other? Perhaps, but now this creates an unpalatable tension with how physics conceptualises ‘what is real’ when it comes to gravity, for physicists (at least when they are being careful and consistent) do not take gravitational force to be real *as a non-inertial force* (anymore than they take geometrical line light-rays or absolute simultaneity to be real). Admittedly physicists’ talk abouts matters of ontology can be imprecise and impressionistic, but the relevance for physics of the conceptual distinction between inertial and non-inertial forces indicates the gravity of the tension in question. The aim to coherently conceptualise gravity at all scales has nothing to do with denying the reality of viruses on the basis of their absence from ‘fundamental’ physics.

To further elaborate on this it is worth recalling the importance of conceptual hygiene regarding the distinction between ‘real’ (or ‘absolute’, or ‘objective’) forces and inertial (‘relative’, a.k.a. ‘pseudo’, or ‘fictitious’) forces within Newtonian mechanics itself. The latter forces, which include Coriolis and centrifugal forces, are always proportional to the mass involved, like gravity is, and they arise from describing the system using a non-inertial frame of reference. In many situations the most natural frame is indeed non-inertial, such as the rotating frame of the our planet for various earthly phenomena associated with the Coriolis force (e.g. weather, or Foucault’s pendulum). There’s no doubt about the theoretical effectiveness of non-inertial forces in various contexts (see any textbook of atmospheric physics for the indispensability of Coriolis force!), and they can capture features of dynamical systems that are undeniably explanatory. Yet when it comes to ontological bookkeeping the ‘fictitious’ status of thus labelled inertial forces is also critical. For what counts as a ‘natural’ frame of reference can be highly contextual, and given that any non-inertial frame gives rise to the corresponding inertial forces, the latter can be multiplied ad infinitum simply through the choice of a coordinate system. Furthermore, it can be shown that the consistency of Newtonian dynamics requires that (real) forces are never acceleration-dependent (Pars, 1965, pp. 11-12).

The aim to carefully conceptualise the qualified sense of ‘reality’ that can be consistently attributed to inertial forces at work in many explanatory contexts is quite different from adopting meta-ontological eliminativism or otherwise. The effective realist’s indiscriminating and inclusive meta-ontology is in tension with this aim. Egg brushes over critical details when he states that “according to the functionalist understanding of ontology […] what it is to be a force is nothing over and above functioning like a force (in all relevant respects).” For what is it to “function like a force”? Coriolis force explains why riverbanks above and below the equator erode more from one side than the other (Baer’s law). On the other hand, inertial work cannot do mechanical work. The overall picture needs to be squared on pain of theoretical inconsistency, and in effort to do that physicists have uncovered important connections between theoretical descriptions. Should realists not respect these widely accepted scientific achievements with their realist commitments?

I think the answer is obviously ‘yes’, and with that in mind I will now finally problematise Egg’s main proposal: effective realism about Textbook Quantum Mechanics.

## TQM: Realism about what?

Egg’s key proposal regarding realism and quantum mechanics is that

“ontology should be informed by our best current theories and that what makes QM one of our best (i.e. empirically most successful) theories is not any of its ontologically kosher (speculative) formulations, but the somewhat messy and recipe-like form in which it appears in textbooks. Hence TQM should be taken ontologically seriously.” (p. 6)

Egg’s conviction is not only that quantum textbooks share an understanding of “uncontroversial examples of scientific achievements that any version of QM must be able to reproduce,” (p. 6) but also that this understanding can furthermore be “taken ontologically seriously” by the scientific realist. And the key to taking TQM ontologically seriously is to construe it in ‘functional’ terms:

“State vectors (or wave functions) codify the behaviour that quantum systems display in virtue of their quantum states in given experimental situations. This is the sense in which the ontology of quantum states is given by what they *do*, namely to bring about specific kinds of behaviour in the quantum systems that are in those states.” (p. 8.)

Egg needs to elaborate on the sense in which quantum states *bring about* observable quantum behaviour (i.e. predicted measurement statistics) in order to fulfil the realist aspiration to justify the theory as an explanatory representation of reality. Pointing to quantum textbooks does not suffice given their notorious ambiguity regarding QM’s representational status. To illustrate, consider Marvin Chester’s *Primer of Quantum Mechanics* (1987), to pick one textbook at random. Chester talks of electron spin as being “twice as effective as is orbital angular momentum in producing a magnetic moment” (p. 154) — ‘producing’ clearly chimes with ‘doing’ — but he also explicitly states that: “There are only two questions that are answered through quantum mechanics: 1. What is the spectrum of possible results in an experiment. 2. What is the probability of finding each result in this spectrum.” (p. 159) Noting the obvious implication that “there are no other questions that one can ask of nature,” it becomes illegitimate to ask for a further clarification of the sense in which the spin ‘produces’ a magnetic moment.

In response to this admonition it is natural to ask, as John Bell (1987) did, why only measurement statistics are thus ‘speakable’ in quantum mechanics, while nothing can be spoken about the reality behind a single round of the Stern-Gerlach experiment? Arguably Bell’s misgivings about the lack of coherent ‘beables’ in TQM have nothing to do with a yen for ‘fundamental’ ontology, and all to do with the inability to extract from the theory any coherent explanatory ontology whatsoever.

The effective realist’s predicament can be illustrated with spin and the Stern-Gerlach experiment. Egg argues at length that “the spin part of the quantum mechanical wave function *refers to a real physical property*” (pp. 11–12, my emphasis). But even if we grant that ‘spin’ refers, and thus that there are true propositions such as “electrons have spin-1/2”, this by itself should not comfort the realist (cf. Stanford, 2015). After all, what realists should care about is not reference per se, but being able to trust theories as good *representations* of the reality behind the appearances. In the case of TQM the worry is not that some future (more ‘fundamental’) theory could replace TQM as an effective recipe for calculating statistical measurement outcomes with undeniable accuracy and reliability. Rather, as Egg anticipates, the worry is that “this kind of persistence of TQM can always be viewed as a purely practical matter without any ontological import,” and that its “mathematical precision only implies ontological precision if a clear interpretation of the mathematical apparatus is given.” (p. 22)

In response to this worry Egg’s effective realist

“rejects the fundamentalist’s requirements on a ‘clear interpretation’: as long as TQM precisely informs us about how quantum systems behave as a function of their spin state […] it yields all the ontological precision one can expect from an effective theory like QM.” (p. 23)

This gets us to the heart of the matter. The call for an ‘interpretation’ of the quantum formalism should not be understood as a request for a ‘fundamental’ ontology, and it absolutely need not spring from a fundamentalist meta-ontology. Rather, what is at stake is *conceptual coherence* of the sort that I highlighted in connection with forces. In my view the fact that “TQM precisely informs us about how quantum systems behave as a function of their spin state” does *not* guarantee the ontological precision we need, anymore than the fact that Newtonian gravity informs us about tidal phenomena guarantees the required ontological precision regarding gravitational force. Ditto regarding the Coriolis force in theories of atmospheric circulation that inform us about wind patterns, etc. In the latter case this is because we lack the desired conceptual coherence overall, and the realist’s commitments should reflect how physics in the grand scheme of things demotes the ontological status of both Newtonian gravitational and Coriolis forces. Similarly, it is the conceptual incoherence of the textbook quantum ‘recipe’, both in its own right and in relation to alternative (internally coherent) formulations of QM, that undermines the realist trust in TQM as yielding the ‘ontological precision’ we should require.

An alternative is to adopt a broadly instrumentalist stance towards the quantum formalism (as many textbooks do), but trying to go beyond such instrumentalism (as many textbooks also inadvertently do!) to explain the working of the Stern-Gerlach apparatus quickly generates theoretical tensions of the sort the realist should pay heed to. Egg maintain’s that “surely TQM gives us some knowledge about what happens in a Stern-Gerlach machine,” but does TQM really explain the experiment’s outputs in a realist manner?^5^ Answering this question requires looking at the textbooks. In lieu of sampling the latter, I will frame my prognosis with reference to Charles Sebens’ (2020) clear synthesis of the standard textbook account.^6^

Sebens begins by highlighting the two aspects of the Stern-Gerlach experiment that call for an explanation. *Discreteness*: “electrons always hit the detector in one of just two possible locations.” *Uniqueness*: “the entire electron [is] found at a single location on the detector.” He then reviews how TQM represents the Stern-Gerlach set-up by mathematically representing the electron with a two-component wavefunction $\chi (\vec {x})$ that evolves according to the Schrödinger equation. The interaction between the wavefunction and the magnetic field $\vec {B}_{S G}$ is incorporated into the Hamiltonian operator $\widehat {H}$:

1 $$ i \hbar \frac{\partial \chi}{\partial t}=\widehat{H}\chi=\left( \frac{-\hbar^{2}}{2m} \nabla^{2}+\mu \vec{\sigma} \cdot \vec{B}_{S G}\right) \chi  $$

Here $\vec {\sigma }$ is associated with the electron’s spin. The incoming electron wavefunction is usually modelled as a Gaussian wave packet that moves through the inhomogeneous magnetic field of the Stern-Gerlach device. After briefly interacting with the magnetic field (for period Δ*t*), the wavefunction can be calculated to have evolved approximately into

2 $$ \chi(\vec{x})=\left( \frac{1}{\pi d^{2}}\right)^{3 \slash 4} \exp \left[\frac{-|\vec{x}|^{2}}{2 d^{2}} - \frac{i}{\hbar} \mu B_{0} {\Delta} t + \frac{i}{\hbar} \mu \eta z {\Delta} t\right]\binom{1}{0}  $$

in the case of *z*-spin up electron.^7^ Sebens describes the significance of this equation:

The $\frac {i}{\hbar } \mu \eta z {\Delta } t$ term […] is critical. This *z* dependent phase oscillation has given our wave packet a non-zero momentum in the *z* direction. As a consequence, the wave packet will move upwards as it evolves under the free Hamiltonian. (p. 14)

The language of a wave packet *moving* due to *interacting* with the magnetic field exemplifies Egg’s conception of effective ontology, “what happens in a Stern-Gerlach machine”. Such language, typical of TQM presentations of the Stern-Gerlach experiment, unavoidably evokes an image of the electron (wave packet) moving, accelerating, interacting, dividing, etc. inside the experimental set-up. All this is suggestive of an effective ontology: surely *something* (whatever its precise ‘nature’ is) is moving and ‘doing’? It is critical to realise, however, that at this point of theorising all we have is mathematics (featuring time and space variables, and thus dynamics) that calls for a physical interpretation beyond the empirical predictions that can be derived from it.

The issue of interpretation arises inevitably when we ask how the mathematics actually explains either *uniqueness* or *discreteness*. At this point we face the quantum measurement problem, indicating the internal incoherence of TQM. The derivation that led to (2) in the case of *z*-spin up electron gives rise to a quantum superposition of the wave packet moving up and down in the case of *x*-spin up electron, in (apparent) contradiction with *uniqueness*. To resolve (or dissolve) the contradiction, different theories of QM (e.g., GRW, Bohmian Mechanics=BM, Many Worlds=MW) say different things about how the mathematics should be interpreted. These theories lead to radically different explanations of both *uniqueness* and *discreteness*.^8^

Egg’s attempt to read an ‘effective’ ontology off textbooks, while rejecting “the fundamentalist’s requirements on a ’clear interpretation”’, leads to theoretical tensions between TQM and the various purportedly coherent theoretical schemes that can explain both *uniqueness* and *discreteness*. To the extent these schemes are regarded as candidate research programmes the realist should acknowledge the potential for tensions comparable to those discussed above in connection with forces. (In the latter case these tensions occur in relation an already accepted body of theory, while in the case of TQM they occur in relation to candidate theories.) Egg denies that these schemes seek to *replace* TQM. He claims that should be rather viewed as ‘more fundamental theories’ that *recover* TQM.^9^ I disagree (in as far as TQM provides an explanatory ontology). The explanatory frameworks are radically divergent, and there is no ‘natural domain’ (in Egg’s sense, cf. §2) where BM, for example, reduces to TQM.

Most obviously, the explanations of *uniqueness* underwritten by GRW, BM, and MW are radically different from one another, while TQM is notoriously unable to explain *uniqueness* at all. For instance, in BM *uniqueness* is explained by the fact that as a *point-* particle the electron can only be at one place at once. By contrast MW explains *uniqueness* by reference to the emergence of dynamically independent branches due to decoherence. Egg (§4.3) notes that the underdetermination regarding wavefunction collapse is not principled, but rather open to empirical investigation. He argues that it does not undermine realist commitment to TQM, but it is entirely unclear to me what *explanatory* commitments can be associated with the latter in relation to *uniqueness*.

There is also tension in the explanations of *discreteness*. Let’s focus on the contrast between TQM and BM. In TQM the whole wave packet for an electron in *z*-spin-up eigenstate moves up due to its interaction with the magnetic field, as per textbook analyses. (These analyses typically involve formal analogies of classical Larmor precession of a magnetic moment about a magnetic field.) That’s all there is to it. In BM the wavepacket is accompanied by the particle that moves up. If *x*-spin-up electron is fed into the same device, the wave packet splits into two components, one going up and one down, and in BM only one of the components is accompanied by the particle, depending on its (unknowable) starting position being either above or below the unique critical trajectory (Norsen, 2014). If the electron is now measured as *z*-spin up, say, this is due to its initial location; the very same electron would have come out as *z*-spin down if the polarity of the magnet was reversed. This exemplifies the contextuality of spin properties. Mathematically the wavefunction is equally involved in both analyses, of course, but its ontological and explanatory status can be very different, with BM in particular being arguably compatible with a purely nomological construal of the wavefunction.^10^ This difference is particularly critical in connection with phenomena involving more than one Stern-Gerlach magnets. Textbook accounts of sequential Stern-Gerlach experiments (in which a *z*-spin up beam is fed into an *x*-spin measurement, and so on) differ radically from how BM accounts for the measurement statistics and the associated Heisenberg uncertainty relations. Ditto for Bell experiments on spin-singlet states. Norsen (*ibid.*) provides a clear discussion of both types of experiments, emphasising how BM explanations bring to the fore the *contextuality* of properties such as ‘being *z*-spin up’, for example. This illustrates how BM would replace TQM’s account of spin in significant ways (in so far as the latter actually tries to explain quantum behaviour in terms of electrons’ property of spin).

The importance of contextuality of spin in BM is emphatically underlined by Norsen:

The key question, though, is precisely whether any such property exists. As has been discussed [by Daumer et al. (1996)], the real lesson to be taken away from examining the pilot-wave perspective on spin is that so-called “contextual properties” (like the individual spin components in the pilot-wave theory) are not properties at all. *They simply do not exist* and there is nothing mysterious about this at all. (p. 346, my emphasis)

The contextuality of spin in BM means that it is not real as a property of quantum particles, full stop. It doesn’t mean that spin is only real as quantum system’s emergent property (like temperature), say, while not being a ‘fundamental property’.

Norsen also rightly calls out the “schizophrenia” of the quantum textbook’s treatment of spin, the conceptual incoherence of the ontological “double-speak” in relation to the spin as explanatory of electron’s interaction with the magnetic field.^11^ The effective realist cannot read the reality of spin and “what happens inside a Stern-Gerlach” magnet from the quantum textbooks without facing up to this lack of coherence, and she furthermore risks conceptual incoherence with more fundamental physics if BM is on the table. Of course, spin is still ‘real’ (in a sense) even in BM, but it is so in a way that is comparable to the ‘fictitious’ forces in classical mechanics.^12^

## Conclusion

I have problematised Egg’s notion of “effective ontology” in general terms and I have illustrated the problem in relation to both quantum mechanics and Newtonian mechanics. I have argued that effective ontology is overly promiscuous and in tension with how physics deals with theoretical inconsistencies by adjudicating ontological commitments in a more fine-grained manner. I leave it to further work to explore the extent to which these issues may or may not apply to other effective realist arguments.^13^ A notable difference between Egg’s effective ontology and the effective realism defended by Williams (2019) and Fraser (2018, 2020) in connection with quantum field theory is that the latter explicitly relies on the effective QFT models’ dependence on energy and length scales. No comparable relationship holds between TQM and BM, GRW, or MW. While Williams’ and Fraser’s realist strategies in the context of QFT are underwritten by specific formal features of effective field theories, Egg’s conception of effective ontology is couched in much broader functional terms that leads to ontological promiscuity.

If the ideology of effective realism does not serve to delineate defensible realist commitments with respect to quantum physics, what alternative options are there? One possibility is to give up on traditional conception of realism as *real*-ism: the idea that realism is a matter of defending knowledge about ‘what is real’. Instead, we can consider construing realism in alternative terms, as justified optimism about quantum theories as representationally successful in ways that we simply haven’t been able to figure out yet (Saatsi, 2019, 2020).

## Footnotes

1 All references to Egg are to this paper.

2 This view shared by scientific realists at large can be contrasted with the “standard account of theory interpretation” that (Williams, 2019, §2) attributes to philosophers of physics. The latter involves interpretive commitments such as: “(1) The theory to be interpreted is assumed to provide a true and exhaustive description of the physical world in all respects, including at all length scales.” (p. 211) This commitment seems overly stringent from the point of view of the scientific realism debate. For example, it does not fit Einstein’s realist argument for atoms on the basis of Perrin’s experiments and his own theorising.

3 Philosophers have of course articulated various views that provide warrant for higher-level ontologies: e.g., Dupré’s (1995) promiscuous realism, Cartwright’s (1999) patchwork realism, and the ‘rainforest realism’ of Ladyman and Ross (2007). On the other side of the meta–ontological smorgasbord there are also hard-core eliminativist positions, such as French (2014).

4 Egg’s brief discussion of the inter–theoretic relationship here glosses over the conceptual discontinuity: “There are […] theoretical accounts of how certain configurations of quarks and electrons can give rise to viruses, but the same is true for the relationship between spacetime curvature and gravitational forces.” As I have indicated it is not the case the GTR “gives rise” to a *non–inertial* gravitational force.

5 The endmost qualification is critical, since arguably a non–representationalist stance towards TQM can accommodate its explanatory achievements to some extent (Healey, 2015). This raises subtle issues about the nature of explanation and explanatory ’black–boxing’, however (Jansson, 2020).

6 See (Sebens, 2020) for references to standard textbooks.

7 The Bohr magneton *μ* captures the strength of the electron’s magnetic moment due to spin. *η* is a constant characterising the inhomogeneity of the magnetic field. *d* is a constant that captures the width of the Gaussian wave packet.

8 Sebens explicitly acknowledges this: “When I say that non-relativistic quantum mechanics can explain these features of Stern–Gerlach experiments, I mean that the theory can do so once it has been formulated in a precise way and the measurement problem has been solved.” (p. 17–18)

9 E.g. “I do not know of any minimalist Bohmian explanation of spin–related phenomena that seeks to replace what TQM teaches about spin. Rather, such explanations seek to recover the TQM account and to provide a firm ontological basis for it.” (p. 14)

10 Egg (§5.2) argues that “any reasonably account of QM needs to admit at least some aspects of the wave function into its ontology” (p. 21). A detailed assessment of his argument is beyond the scope of this paper, but suffice to say that in my view it relies on metaphysical presuppositions (regarding action at a distance, and the relationship between ontology and dynamics) that I do not find compelling on the basis of empirical evidence that I take to support realism about quantum mechanics.

11 Norsen traces this to textbook authors’ need — despite their occasional instrumentalist admonitions — for “some sort of visualizable picture of what, physically, the mathematical formalism describes, or they simply cannot keep track of what in the world they are talking about.” (p. 347)

12 I have focused here on BM for dialectical purposes only, not as an argument in favour of this research programme. More generally, it can be argued that the varied landscape of quantum foundations presents an underdetermination challenge that transcends the three currently dominating programmes (Saatsi, 2019).

13 Egg draws inspiration from Cordero (2001), as well as Porter Williams (2019) and James Fraser (2018, 2020). Laura Ruetsche (2018) critically discusses the latters’ effective (‘renormalization group’) realism, highlighting a challenge from Newtonian gravity. Wallace (2012) argues, in connection with Everettian quantum theory, for a meta-ontology of ‘real patterns’ as determining which macro-objects in science are real. Relatedly, Ladyman and Ross (2007) defend a ‘real pattern’ ontology (and an associated idea of scale-relativity of ontology) as a form of ontological structural realism.
